# HerpSexDet: the herpetological database of sex determination and sex reversal

**DOI:** 10.1038/s41597-023-02268-y

**Published:** 2023-06-13

**Authors:** Edina Nemesházi, Veronika Bókony

**Affiliations:** 1 Konrad Lorenz Institute of Ethology, Department of Interdisciplinary Life Sciences, University of Veterinary Medicine Vienna, Savoyenstr. 1a, A-1160 Vienna, Austria; 2 Veterinary Medical Research Institute, Hungária Krt. 21, H-1143 Budapest, Hungary; 3 Department of Evolutionary Ecology, Plant Protection Institute, Centre for Agricultural Research, Eötvös Loránd Research Network, Herman Ottó u. 15, H-1022 Budapest, Hungary

**Keywords:** Evolutionary developmental biology, Evolutionary ecology

## Abstract

Wildlife exhibits various sex-determination systems where sex chromosomes and environmental temperatures may both contribute to individual sexual development. The causes and consequences of this variability are important questions for evolutionary ecology, especially in light of ongoing environmental change. Amphibians and reptiles are emerging as a key group for studying these questions, with new data accumulating acceleratingly. We collected empirical data from earlier databases, reviews and primary literature to create the most up-to-date database on herpetological sex determination. We named our database HerpSexDet, which currently features data on genetic and temperature-dependent sex determination as well as reports on sex reversal for a total of 192 amphibian and 697 reptile species. This dataset, which we will regularly update in the future, facilitates interspecific comparative studies on the evolution of sex determination and its consequences for species-specific traits such as life history and conservation status, and may also help guiding future research by identifying species or higher taxa that are potentially most enlightening for the study of environmentally driven sex reversal.

## Background & Summary

The diversity of sex-determination systems is stunning across eukaryotic organisms, and the causes and consequences of this diversity are important questions in evolutionary ecology^1^. This importance is heightened by contemporary human-induced environmental changes, because sex determination is often sensitive to environmental conditions, and thus the type of sex-determination system can influence how environmental change affects population genetics, demography, and extinction risk^2–5^. In gonochoristic taxa, two widespread systems are genetic and environmental sex determination, the latter often meaning temperature-dependent sex determination. Although these two types were conventionally considered mutually exclusive, they are now seen as two theoretical ends of a continuum, where high or low temperatures can override the sex otherwise defined by sex chromosomes^6^. As a result, sex reversal can occur, where genetically female individuals develop male gonads or the other way around. These topics have been attracting scientific attention at an accelerating rate in the past decade^7–10^, with ectothermic vertebrates emerging as a prominent “model taxon” due to their high variability in sex determination both across and within species^11,12^.

Nearly 10 years passed since the publication of the Tree of Sex database, the most comprehensive collection of sex-determination systems across vertebrates, invertebrates and plants^13^. Since then, Tree of Sex has been used for studying many research questions (e.g.^8,10,14–16^), and the “genomics era” has yielded exponential growth in novel data on sex-determination systems and revisions of molecular taxonomy; there is thus need for an up-to-date source of comparative data on sex-determination systems. Reflecting this need, two recent databases haven been published on amphibian karyotypes^17^ and sex-determination mechanisms in turtles^18^. Several authors also complemented the Tree of Sex database with additional sex-determination data for their own study objectives (e.g.^8,10,14,16^). Our goal was to gather the currently available data on the type of heterogamety (genetic sex determination, GSD) and temperature-dependent sex determination (TSD) across amphibians and reptiles, to provide a comprehensive database for future studies. Given the importance and recent growing interest in sex reversal^8,19^, we also collected all available data in these taxa on experimentally proven temperature-induced sex reversal and the occurrence and direction of sex reversal in wild populations. Because sex determination may be related to polyploidy^20,21^, we also collected data on the occurrence of polyploidy. We did not include fish, because the high diversity of sex determination in this class of ectothermic vertebrates is complicated by variation among artificially selected strains in economically relevant species, sequential hermaphroditism, and non-thermal triggers of environmental sex determination^9^, calling for a future database focusing on fish.

Altogether, HerpSexDet features sex-determination data on 192 amphibian and 697 reptile species, and two amphibian hybrids (891 rows and 32 columns; Tables 1, 2). Specifically, our dataset contains data on the type of heterogamety for 189 amphibian and 541 reptile species (vs. 99 and 351 species in Tree of Sex, respectively), and data on the effects of temperature on sex determination in 18 amphibian and 171 reptile species (vs. 0 and 134 in Tree of Sex, respectively). Besides the type of TSD, HerpSexDet includes detailed information on sex ratios at low, intermediate and high temperatures as well as temperature thresholds based on empirical data in 18 amphibian and 135 reptile species.

**Table 1:** Short description of data collected for the HerpSexDet database.

Category	Variable name	Short description
GSD	GSD_type	Type of heterogamety. Usually XX/XY, ZW/ZZ, complex XX/XY or complex ZW/ZZ.
	Sex_chromosome_karyotype	Have sex chromosomes been identified by karyotyping? Values: homomorphic or identified.
	Size_difference	Size difference between the sex chromosomes is indicated by “yes”, no difference is indicated by “no”. Note that this information was collected non-systematically in this first version of HerpSexDet.
	Larger_chromosome	The relatively larger sex chromosome (X, Y, Z, or W).
TSD	TSD_type	TSD reaction norm. Usually MF (type Ia), FM (type Ib), or FMF (type II).
	TSD_type_detailed	More detailed categorization of the TSD reaction norm, including information on even sex ratios.
	Low_temperature_sex	The phenotypic sex in excess at the lower range of investigated temperatures: male, female, or even.
	Low_temperature_threshold	Temperature (°C) below which biased sex ratio is produced.
	Intermediate_temperature_sex	The phenotypic sex in excess at the intermediate range of investigated temperatures: male, female, or even.
	High_temperature_sex	The phenotypic sex in excess at the higher range of investigated temperatures: male, female, or even.
	High_temperature_threshold	Temperature (°C) above which biased sex ratio is produced.
Sex reversal	Thermal_sex_reversal_proven	Is there experimental evidence for thermally induced sex reversal? Yes or no, if it was studied.
	Sex_reversal_in_nature	Combinations of sexual genotypes and phenotypes found in wild populations.
Other	Polyploid	Information on the occurrence of polyploidy.

Detailed description including further column names for references, notes and curators is available at^39^.

**Table 2:** Distribution of sex-determination and sex-reversal data across taxonomic orders in the HerpSexDet database (the number of species is shown).

Class	Order	GSD	TSD^1^	GSD and TSD^1^	Thermal sex reversal proven	Sex reversal in nature
Amphibia	Anura	135	11^2^	7	2	6
	Caudata	52	7 (1)	6 (1)	4	—
	Gymnophiona	2	—	—	—	—
Reptilia	Crocodylia	—	14	—	—	—
	Rhynchocephalia	—	1	—	—	—
	Squamata	509	58 (27)	14 (6)	3	2
	Testudines	32	98 (12)	1 (1)	—	—
*Sum*		*730*	*189*	*28*	*9*	*8*

^1^The number of species for which the existence of TSD is uncertain due to lack of reliable data (variable “TSD_type”, value “TSD uncertain”) is indicated in parentheses next to the total number of species with either certain or uncertain TSD.

^2^9 species and two hybrids.

We hope that HerpSexDet and its future updates will significantly contribute to sex-related evolutionary ecology research worldwide. Sex-determination systems have been replacing each other frequently during ectotherm evolution^12,14,22,23^, and sex reversal has been suggested to play a role in this process^2,24–26^. However, the evolutionary forces and molecular mechanisms underlying these turnovers are poorly understood. Recent works suggested that aging rates may be higher in the heterogametic sex^27,28^ and species with different types of heterogamety might respond differently to anthropogenic environmental changes^3,4,8,29^. Therefore, our dataset may be of use for comparative studies on the evolution of sex determination, physiological traits and habitat adaptations. For example, HerpSexDet could be used together with other databases to assess questions on temperature-related developmental plasticity and heat tolerance^30,31^, or to evaluate if life-history traits^32,33^ differ by sex-determination systems. We expect that such studies will significantly improve our understanding of fundamental evolutionary processes as well as the potential consequences of the ongoing anthropogenic changes worldwide.

## Methods

### Data collection

**Fig. 1:**
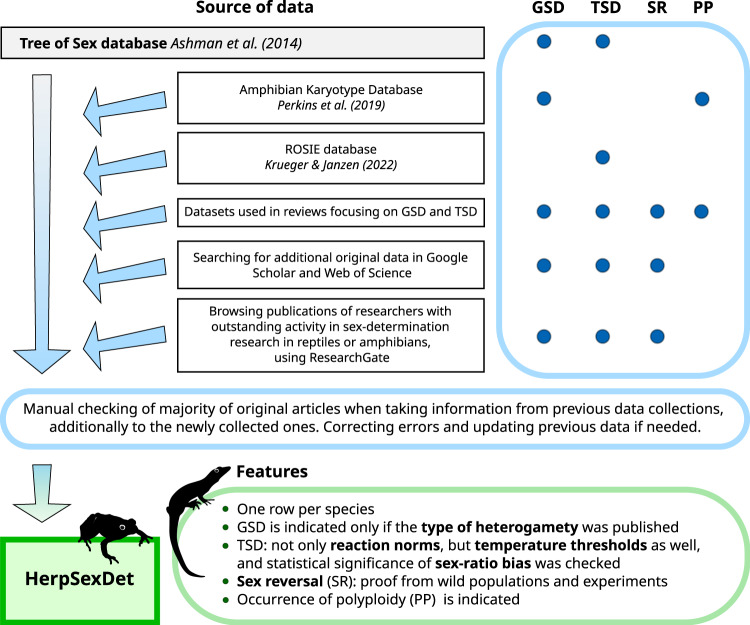
Workflow of data collection and key features of HerpSexDet. The Tree of Sex database provided a baseline for species-specific data on genetic (GSD) and temperature-dependent sex determination (TSD), and was double-checked, complemented, and updated with new data. HerpSexDet also provides further details on TSD, including temperature thresholds, as well as sex-reversal (SR) data from wild populations and the occurrence of polyploidy (PP).

We collected data on sex determination in herpetofauna by relying on and revisiting previous data collections and adding more recent data (Fig. 1). As a starting point, we incorporated species-specific data on the type of heterogamety and the type of TSD reaction norm for amphibians and reptiles from the Tree of Sex database^13^. In case of those species for which data were shown in multiple rows in Tree of Sex (e.g. the same species occurring with different names, different subspecies, or different data sources), we merged the information into a single row, to represent the consensus data for each species. We preferred the one-row-per-species format because information on within-species variation in sex determination is very limited. However, in those few cases where we found such information, we noted it in the database (in variables “GSD_note”, “Size_note”, or “TSD_note”). Next, we collected sex-determination data for additional species, as well as more up-to-date data for the species already represented in the Tree of Sex database. For this purpose, we overviewed sex-determination data in two additional databases^17,18^ as well as the datasets used in review articles and book chapters focusing on sex determination or the temperature effects on sexual development in amphibians or reptiles^8,14,34–38^. We aimed to double-check the original sources cited in these collections and Tree of Sex, although this did not always happen. For example, in several cases, we could not identify the reference given in Tree of Sex, so we took these references unchanged, and noted this fact in the “Comment” column of the reference table^39^. Furthermore, we double-checked the cases where we found contradictions between the older and more recent data. During these double-checks, we revised the information that was given in the earlier databases whenever necessary. Finally, we searched for additional species-specific data in primary literature using Google Scholar (https://scholar.google.com) and Web of Science (https://www.webofscience.com). In the search phrases, we combined *taxon* names (class or order) with one of the following keywords: *sex determination*, *heterogamety*, *sex chromosome*, *XY*, *ZW*, *TSD*, or *temperature-dependent sex determination*. After reading the title and abstract of each article, we decided whether to read the article and extract data from it, based on whether the paper reported species-specific information either on the type of heterogamety or on the effect of temperature on sex determination or sex ratios. As we found that certain researchers were contributing especially actively to sex-determination research in reptiles or amphibians, we also browsed their articles on ResearchGate (https://www.researchgate.net). Furthermore, we collected data on the occurrence of sex reversal in wild populations as we encountered such reports (we did not do a targeted search for this because sex reversal in free-living herpetofauna is a young field of research which we have been continuously monitoring). After we finished collecting GSD data on the 21^st^ of December 2022, we performed a final search in Google Scholar for TSD data in those families for which GSD data were present in our dataset, using search phrases combining *temperature*, *sex ratio*, and *incubation* (for reptiles) or *larvae* or *tadpoles* (for amphibians). We collected data on the occurrence of polyploidy from a database dedicated to amphibian karyotypes^17^ and a review on reptiles^40^.

### Standards for data inclusion

We extracted data from each source manually. We extracted data with caution and evaluated the text of the article overall before including the data in our database, keeping only those records that provided reliable information on the sex-determination system in a species (see the paragraphs below for more specific criteria). When we found contradiction between reports in well-established journals and grey zone publications or mentions of non-accessible resources, we disregarded the latter.

We included GSD data only for those species in which the type of heterogamety (“GSD_type”, see Table 1) was identified: mainly by karyotypes or sex-linked molecular markers, but in some studies in the previous century, heterogamety was assessed based on breeding experiments following artificial sex reversal. Species for which GSD was assumed only based on an observed lack of temperature effects on sex ratios or anecdotical information were excluded from our database. Based on our knowledge on related works, we judged that information on sex-linked DNA loci mapped to a reference species´ genome was available only for a fraction of species with identified sex chromosomes. So, because we assumed that chromosome identity for most species would not be comparable with that of others, we did not indicate which chromosome pair is named as the sex chromosome in each species. Instead, we indicated if the sex chromosomes were identified (variable: “Sex_chromosome_karyotype”, as described in Table 1). The published chromosome numbers can be found in the references for GSD.

If sex-ratio data were available for a species from thermal manipulation experiments, we evaluated TSD by testing whether the ratio of male and female offspring deviated significantly from 1:1 at each applied temperature within each study by performing binomial tests. When significantly biased sex ratio was found at some temperature(s) but not at other(s), we categorized the species into the type of TSD reaction norm that best fit the data (variable “TSD_type”) and we recorded the temperature thresholds below and/or above which sex-ratio bias was significant. Because statistical tests of sex ratios are not reliable at small sample sizes, we added a warning on small sample sizes whenever appropriate (variable “TSD_note”) and marked uncertain data by question marks (see metadata at^39^). For a few TSD species where many studies were done at many different temperatures, we used the estimated boundaries of the transitional range of temperatures that produce both sexes^41^ (see also metadata at^39^). When there was not enough information for formally assessing TSD, e.g. because the original data were not accessible, we categorized the species as “TSD uncertain”.

We indicated the presence of sex-reversed individuals in nature only if it was proven by mismatching sexual genotypes and phenotypes in free-living animals (variable: “Sex_reversal_in_nature”). Potential occurrence of sex reversal in further species was also suggested earlier, based on findings that both GSD and TSD was detected in the same species, although not in the same individuals^19^. We did not include such circumstantial data as evidence for sex reversal in our database (Table 2). However, by combining information present in our database (i.e. if species in the taxon feature both GSD and TSD), researchers may get information on the suggestive occurrence of temperature-induced sex reversal, even if it has not yet been reported in their species of interest (see Table 2); this may help identifying taxa worthwhile for future studies.

### Taxonomy

We extracted species lists manually from two taxon-specific databases^42,43^. Using these lists, we searched for species names present in our dataset and added taxonomic classification (family, order, class) to them using a custom code (see Supplementary File 1). If a species could not be identified automatically, we corrected the entry manually after searching for relevant synonyms on NCBI Taxonomy (https://www.ncbi.nlm.nih.gov/taxonomy). To enable the identification of species names that had been used in our data sources, we listed synonyms as well as subspecies names in the “Synonym” variable. Our dataset contains two rows with TSD data on amphibian hybrids, because TSD data were available in the literature only for these two hybrids within their respective taxonomic families.

## Data Records

The database consists of three files. Data and references are included in two separate text files, while detailed metadata for the database is provided as a pdf file in the Figshare repository^39^. The most important variables are listed and briefly explained in Table 1, but for detailed information see the metadata file.

The database is provided as tabulator-separated plain text (character encoding: UTF-8) named HerpSexDet_v1-1.tsv. Since all data (except for the detailed references) are incorporated in a single file, usage of the database is straightforward. For example, users can simply import the whole dataset to R^44^, and based on the metadata file, they can easily decide which variables are relevant for their study. In the main database file, references are indicated as identifiers (numbers in square brackets), and the full citation for each identifier is available in the References_for_HerpSexDet_v1_1.tsv file. To help users reading our database, we also provided a readme file and an example R code^39^.

## Technical Validation

Data were extracted manually from the referred sources. Subsets of both the GSD and TSD data records were evaluated by both authors, and in most cases the authors agreed in data entries and categorizations. In those few cases when inconsistency occurred, decision was made based on double-checking the original sources and mutual agreement of the two authors.

We encourage researchers to let us know if they find any error in our dataset or if they publish new sex-determination data that should be included in future versions of HerpSexDet.

## Usage Notes

References for GSD, TSD, sex-reversal data from nature, occurrence of polyploidy and taxonomy are listed under five respective variables: “GSD_reference”, “TSD_reference”, “Sex_reversal_reference”, “Polyploid_reference” and “Taxonomy_reference”. Each variable may contain the identifiers of multiple references, separated by commas. Full citations for the reference identifiers are listed in the References_for_HerpSexDet_v1_1.tsv file. To help the users find and check our data sources upon interest, we indicated the DOI identifier of each source if available. Otherwise, we aimed to provide the most unequivocal reference that was possible.

We recommend users to check for updates on https://hsd-project.eu before starting a new analysis based on HerpSexDet, because we are planning to release updates under new version numbers on a regular basis.

## Supplementary information

Supplementary File 1

## Data Availability

The custom code that we used for taxonomic identification is available as Supplementary File 1. Additionally, an example code for reading our database in R is provided at^39^.
